# Evolution of native defects in ZnO nanorods irradiated with hydrogen ion

**DOI:** 10.1038/s41598-019-53951-3

**Published:** 2019-11-22

**Authors:** Tengfei Wu, Aiji Wang, Li Zheng, Guangfu Wang, Qingyun Tu, Bowen Lv, Zilin Liu, Zhenglong Wu, Yinshu Wang

**Affiliations:** 1 Department of Physics, Beijing Normal University, Beijing, 100875 China; 2 College of Nuclear Science and Technology, Beijing Normal University, Beijing, 100875 China; 3 Analytical and Testing Center, Beijing Normal University, Beijing, 100875 China

**Keywords:** Nanoscale materials, Nanowires, Experimental nuclear physics

## Abstract

This work reports the study on the evolution of native defects in ZnO nanorods irradiated with hydrogen ion. ZnO nanorod arrays grown vertically on silicon substrates were irradiated by 180 keV H^+^ ions to a total fluence of 8.50 × 10^15^ ions/cm^2^. The X-ray diffraction spectra, photoluminescence spectra before and after irradiation and the real-time ionoluminescence spectra of the nanorod arrays during the irradiating process were measured. Formation and evolution of defects during H^+^ ion irradiation and effects of irradiation on the crystal structure and optical property were studied. Blue shift of exciton emission, shrink of lattice c and improvement of the crystallinity of ZnO nanorods after irradiation were observed. Simple surface passivation of the nanorods could improve the radiation resistance. Formation and evolution of the defects during H^+^ ion irradiation could be clarified into four stages and the related models are provided.

## Introduction

ZnO has a wide bandgap of 3.37 eV at room temperature and a higher exciton binding energy (60 meV), which has attracted much attention for several decades because of its potential applications in UV detectors, lasers, transparent electronics, piezo-phototronics, solar cells and so on^1,2^. There are various native defects in ZnO such as oxygen vacancy (*V*_*O*_), zinc vacancy (*V*_*Zn*_), oxygen interstitial (*O*_*i*_), Zn interstitial (*Zn*_*i*_), Zn at V_O_ (*Zn*_*O*_), O at V_Zn_ (*O*_*Zn*_) and their pairs or complexes^3–6^. The as-grown ZnO usually exhibits n-type which is traditionally attributed to the native defects such as *Zn*_*i*_, *V*_*O*_, *Zn*_*O*_ and H related shallow defects^3,7,8^. However, the electrical behaviors and origin of the luminescence related with native defects are still in debate. Until now, difficulties still exist in the formation, evolution and control of the native defects, especially in controlling the concentration of native defects during growth under equilibrium condition^6^.

Ion implantation is an attractive tool for semiconductor processing such as doping and isolation, creating controllable defects through non equilibrium^9–11^. There are many reports on the effects of native defects on ZnO properties investigated by ion implantation^6–9,12–23^. Compared with other ion implantation, H ion implantation is still an active topic because H plays a significant role in conductivity of ZnO and it is unavoidably introduced during material growth and processing^6,7^. Most studies concentrate on H ion implantation effects on properties of bulk ZnO crystals^7,15–18^. Heinhold *et al*. investigated H related defects in bulk ZnO and ascribed lithium-hydrogen defect complexes *Li*_*Zn*_*-H*_*O*_, *Al*_*Zn*_*-H*_*O*_*-Li*_*Zn*_, and *V*_*Zn*_*-H* as donors^7^, while Kaida *et al*. attributed the shallow donors to H interstitials^14^. Chan *et al*. observed a lattices elongating of ZnO along c-axis and stabilization of H by defect complexes, such as H_O_ and/or Li_Zn_-OH^15^. They also observed a more efficient damage recovery in the samples implanted with higher dose. Empizo *et al*. observed a suppression of visible emission and a recovery of the photoluminescence of bulk ZnO after being irradiated with H or D ions without any treatment at room temperature^16^. Obviously, dynamic process of ZnO during ion implantation would affect the defect stability and the crystal properties. Thermal annealing effect of ZnO nanorod arrays during photoluminescence (PL) measurement has been reported^19^, indicating that the nanostructures are more sensitive to dynamic annealing. Nanorods/nanowires represent an exclusive and sensitively system for analyzing ion implantation phenomena and bring about significant changes in their properties^11,24^.

Nanostructures of ZnO have attracted much attention in recent years owing to their peculiar and unique properties such as larger specific surface area, more flexibility, obvious quantum effects and so on^1^. There are several reports on irradiation effects of ZnO microstructures and nanostructures^21,25–27^. Botsch *et al*. observed a persistent spin helix in H^+^ ion implanted Li-doped ZnO microwires^21^. They attributed Rashba coupling to the formation of interface in the (10$\bar{1}$0) plane resulted from the Li dopant which stabilize the *V*_*Zn*_ and *V*_*Zn*_*-OH* complexes produced by the H^+^ ion implantation. Ranjith *et al*. observed a surface oxygen-deficiency of ZnO nanorods after irradiated by swift heavy ions^24^. Dee *et al*. observed the transformation of ZnO nanowires from wurtzite structure into a disordered amorphous structure irradiated with 70 keV H^+^ ions to a dose of 2 × 10^17^ ions/cm^2^, which is much lower than that in bulk ZnO^25^. Anyhow, evolution of defects during ion irradiation, especially at the early stage of irradiation is still unclear. Beam-induced luminescence (IL) is a valuable tool for monitoring real-time information during the irradiation, such as the pre-existing defects, the formation of trap centers and their evolution with ion fluence^28–31^. There are only few reports on IL spectra of a bulk ZnO single crystal irradiated by energetic ions^9,13^. Until now, there are seldom reports on IL spectra of ZnO nanostructures.

In this work, ZnO nanorod arrays were grown by the solution method and then irradiated with 180 keV H^+^ ions. Formation and evolution of defects were investigated systematically by real-time IL spectra, combined with X-ray diffraction (XRD) and photoluminescence (PL) analyses.

## Results and Discussion

### Irradiation effects of ZnO nanorod arrays

**Figure 1:**
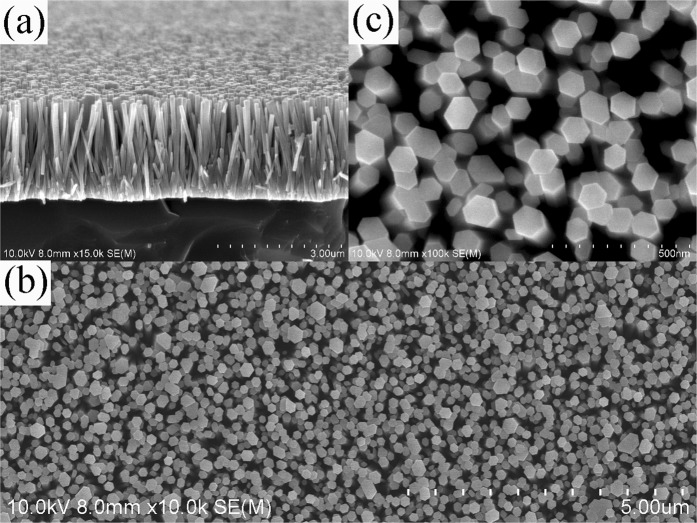
SEM images of the as-grown ZnO nanorods grown on Si substrates. (**a**) cross-sectional view (**b**) plan-view (**c**) enlarged plane view.

**Figure 2:**
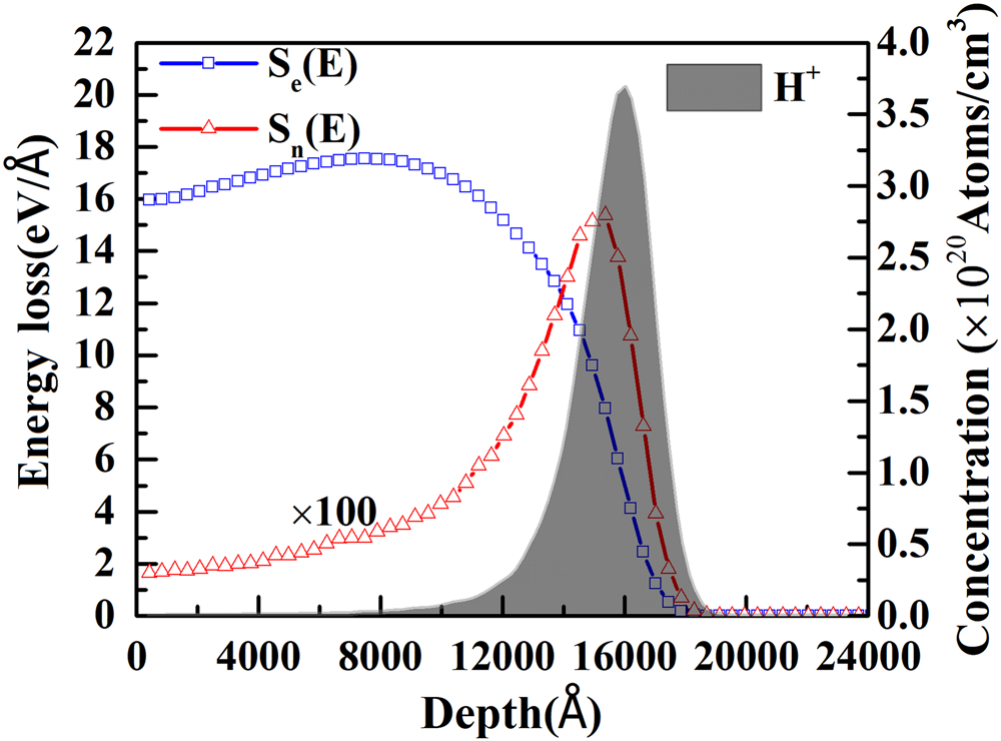
The electronic energy loss (Se), nuclear energy loss (Sn) of 180 keV H^+^ ions and H distribution within the ZnO nanorod layer calculated with SRIM-TRIM (2013).

Figure 1 shows the surface morphology and cross-sectional SEM images of the ZnO nanorods grown on silicon substrates. High density and uniform distributed nanorods with well-defined hexagonal facets were grown vertically on the substrates. Diameters of the nanorods are from 80 to 120 nm and the length of the nanorods is about 2.40 μm measured from the cross-sectional image. The morphologies of the nanorods before and after the ion implantation are similar. The electronic energy loss (Se(E)), nuclear energy loss (Sn(E)) and the concentration of the H atoms in the ZnO nanorod layer were simulated by SRIM-TRIM (2013) code and are shown in Figure 2. The electronic energy loss of H^+^ ions distributes almost uniformly within 1.3 μm and nuclear energy loss is mainly distributed in a narrow region around depth of 1.5 μm. Otherwise, the electronic energy loss dominates and is much higher than that of the nuclear energy loss along most of the ion path. The ion range of H^+^ ion is around 1.603 μm, which is shorter than the length of the nanorods (2.40 μm), indicating that the implanted H ions would be stopped in the ZnO nanorods.

**Figure 3:**
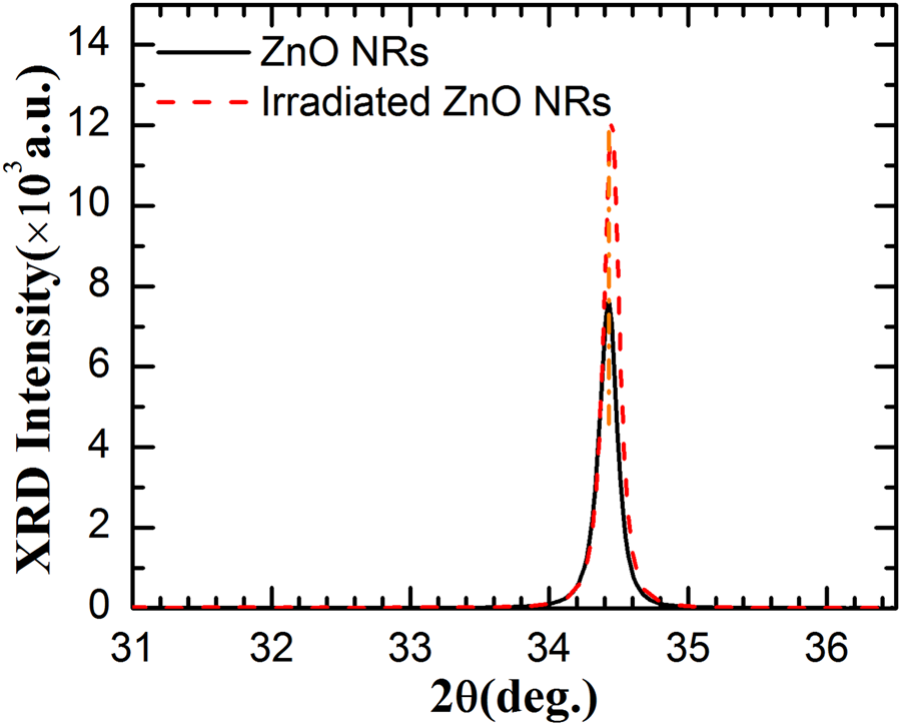
XRD patterns of ZnO nanorod arrays before and after H^+^ ion irradiation.

To investigate the effect of H^+^ ion irradiation on the crystal properties of ZnO nanorods, XRD spectra of the nanorods were measured before and after irradiation. Figure 3 shows XRD patterns of the ZnO nanorods before and after H^+^ ion irradiation. Only (002) plane diffraction is observed, indicating that the nanorods grow along c-axis. The diffraction peak of the as-grown nanorods locates at 2θ = 34.43° with a FWHM of 0.168°, indicating that ZnO nanorods have good crystallinity. After the irradiation, the diffraction peak shifts to a higher angle of 2θ = 34.45°. Meanwhile, the diffraction intensity increases and FWHM decreases to 0.150°, which means that the irradiation results in a shrink of the lattice parameter c and improvement of the crystallinity of the nanorods. Nagar *et al*. observed a similar compression of lattice c in PLD deposited ZnO films^27^. However, Chan *et al*. observed a elongation of the lattice c of bulk ZnO single crystals after H^+^ ion implantation, but most of the strain in the deformed layers could be annealed out after heat treatment at 873~1073 K^15^. The implanted H^+^ ions lose their energy in ZnO nanorods through nuclear collisions and electronic energy loss. Nuclear collisions would result in the formation of point defects such as vacancies, interstitials, etc. by displacement of the lattice atoms, which would degenerate the nanorod crystallinity. The electronic energy loss would excite electrons and phonons, and ionize target atoms in the nanorod along their paths. Then the energy would be released in the form of heat and photons, which could result in annealing of defects and act as the excitation source for the IL spectra^13^. Bulk ZnO has excellent thermal conductivity. Nanorod arrays in this work stand vertically on the surface and there are separate regions between most of nanorods. The heat produced by ions could not efficiently transfer between nanorods. Under the same irradiation fluence, the temperature of the nanorod arrays would be much higher than that of the bulk ZnO. The defects produced by implantation could be annealed more efficiently in ZnO nanorod arrays than that in bulk ZnO. Otherwise, *Zn*_*i*_ and *O*_*i*_ produced by H^+^ ions are sufficiently mobile in the temperature of 290~325 K^8^, while *V*_*Zn*_ and *V*_*O*_ are much stable^4^. The diameters of ZnO nanorods are 80~120 nm. The movable interstitials could diffuse easily to the nanorod surfaces. Then the remaining vacancies would be compensated by newly produced interstitials. H interstitials are also easy to move and the implanted H atoms could be stabilized by *V*_*Zn*_, *V*_*O*_ and their complex^7,17,19^. All this would result in a shrink of the lattice and improvement of the crystallinity of ZnO nanorods as that shown in Figure 3.

**Figure 4:**
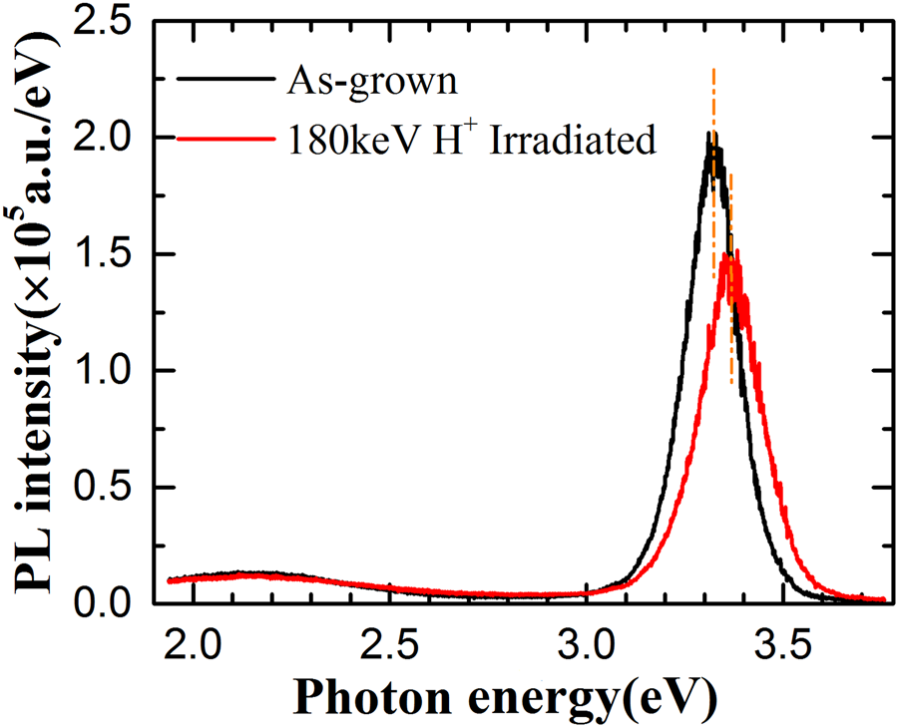
PL spectra of ZnO nanorods before and after H^+^ ion irradiation.

To investigate irradiation effects on the nanorod optical properties, PL spectra of the nanorods were measured before and after irradiation and are shown in Figure 4. Before irradiation, the ZnO nanorods have a strong UV emission at 3.32 eV related with exciton emissions and weak emission at 2.15 eV related with defects. After irradiation, the intensity of UV emission decreases while the intensity of defect related emission is unchanged. Meanwhile, the UV emission peak shifts to 3.36 eV. The weakening of the UV emission is mainly due to the generation of non-radiative recombination centers during the ion implantation. Wang *et al*. observed a blue shift of UV emission of ZnO nanowires excited by a laser with high power. They attributed it to the reduction of the built-in electric field induced by excited carriers^32^. However, a red shift of the UV emission was observed in ZnO nanorods grown with similar method to this work^19,33^. Alvi *et al*. observed a similar blue shift of UV emission in ZnO nanorods/p-GaN light emitting diodes irradiated by helium-ion bombardment^26^. They attributed the shift to compression strain in ZnO nanorods. Compression strain would result in widening of semiconductor bandgap^34^. In this work, compression is also observed as that shown in Figure 3. The blue shift of the UV emission in Figure 4 could be ascribed to the compression of ZnO nanorods after irradiated with H^+^ ions.

### Evolution of the real-time IL spectra of ZnO nanorod arrays

**Figure 5:**
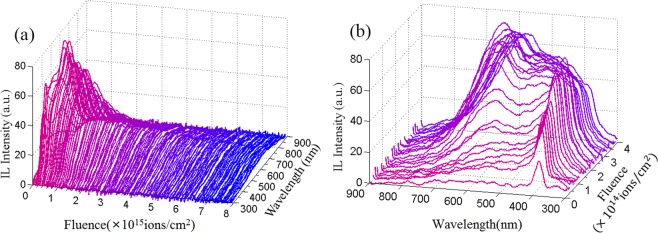
(**a**) IL spectra of ZnO nanorods irradiated by 180 keV H^+^ to different ion fluences (**b**) IL spectra at the beginning stages of irradiation with ion fluence from 0 to 4.8 × 10^14^ ions/cm^2^.

**Figure 6:**
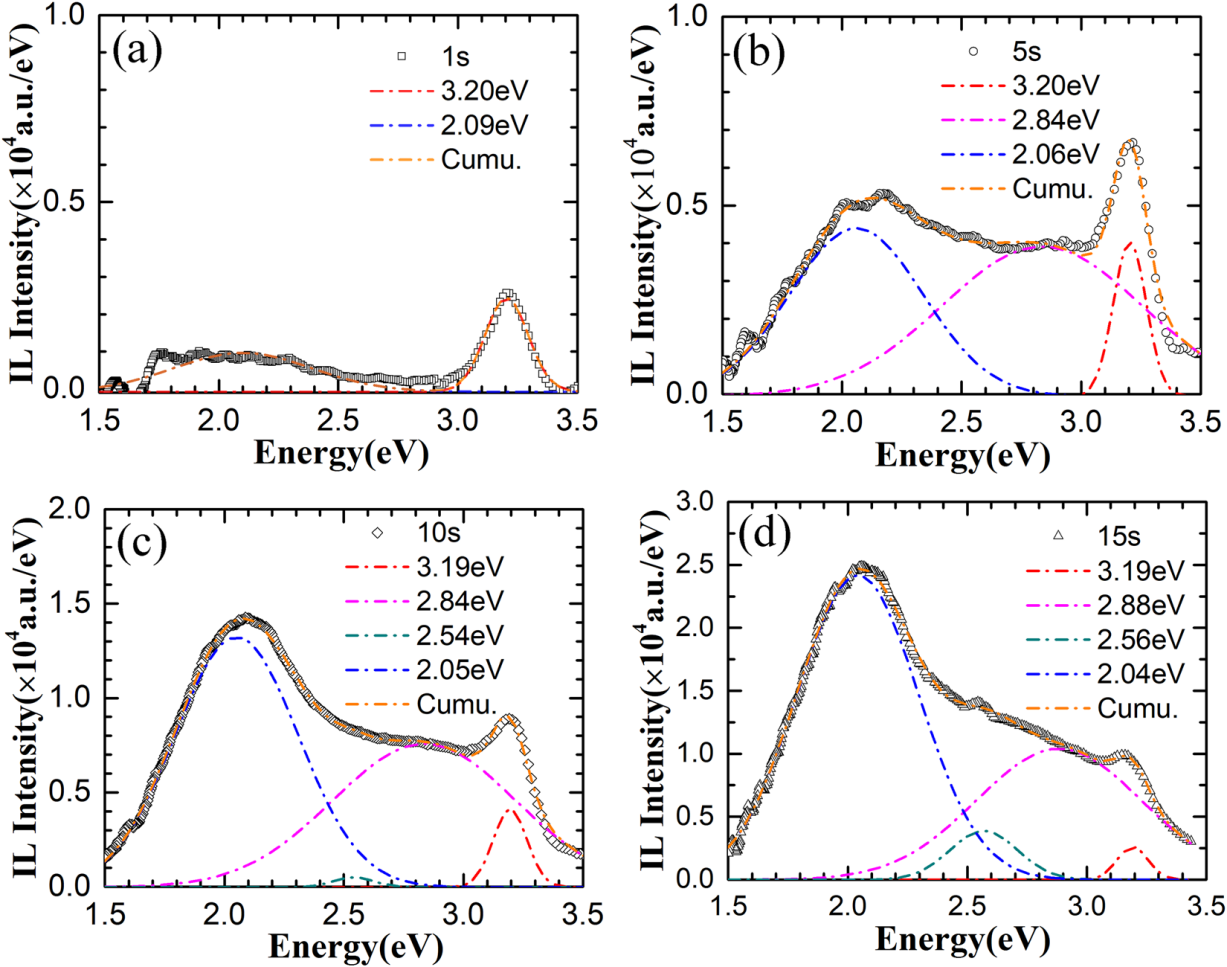
Gaussian decomposition of the real-time IL spectrum of the ZnO nanorods after irradiation by 180 keV H^+^ ions for (**a**) 1 s, (**b**) 5 s, **(c**) 10 s, (**d**) 15 s, the corresponding H^+^ ions fluences are 1.6 × 10^13^, 8.0 × 10^13^, 1.6 × 10^14^, 2.4 × 10^14^ ions/cm^2^, respectively.

To investigate the evolution of the irradiation effects, real-time IL spectra of ZnO nanorods were collected during H^+^ ion implantation. The evolution of the spectra with the increasing of H^+^ ion fluence is shown in Figure 5(a). Both of the UV and visible emission and their relative intensity vary dramatically with the increasing of the irradiation fluence, especially at the beginning stage with the irradiation fluence lower than 2 × 10^14^ ions/cm^2^. With a further increase in H^+^ ion fluence, the UV emission decreases. Meanwhile, the visible emission decreases gradually. To see the variation of the emission at early stage clearly, typical spectra of ZnO nanorods irradiated with different fluence lower than 4 × 10^14^ ions/cm^2^ are shown in Figure 5(b). Primitively, only UV emission centered at 387 nm and very weak visible emission at 593 nm are observed. Once the nanorods are irradiated to a fluence of 8 × 10^13^ ions/cm^2^, both of the UV emission and visible emission enhance dramatically. Otherwise, the IL spectrum line shape changes obviously. This suggests that activation/formation and quenching/passivation of emission centers are very sensitive to H^+^ ion fluence at the early stage. To investigate the evolution of emission centers clearly, representative IL spectra after irradiated for different H^+^ ion fluences are fitted by multi-peak Gauss functions. IL spectra after irradiation for 1 s, 5 s, 10 s and 15 s, corresponding the ion fluence of 1.60 × 10^13^, 8.00 × 10^13^, 1.60 × 10^14^ and 2.40 × 10^14^ ions/cm^2^ are shown in Figure 6. Note that these IL spectra have been converted from wavelength space (originally in units of I(λ)dλ) to energy space (I(E)dE), which is required to represent the relative photon yields between different emission peaks. At the beginning stage of ion fluence of 1.6 × 10^13^ ions/cm^2^, only an obvious peak at 3.20 eV and a relatively weak band centered at 2.09 eV are observed. Comparing with PL spectra in Figure 4, UV emission center shifts from 3.32 to 3.20 eV. UV emission energy detected by IL spectra in bulk ZnO is around 3.22~3.26 eV, which is also lower than that detected by PL spectra and the emission has been attributed to near band edge emission^12,16^. IL spectra were measured during ion irradiation and the temperature of the samples would be higher than room temperature, which would result in narrowing of the bandgap and lead to a red shift of the emission. On the other hand, weak excitation during IL collection would result in the dominance of near band edge emission and vanishing of exciton emission. Emission at 2.09 eV has been ascribed to *V*_*Zn*_ cluster related transitions by Dong *et al*. based on positron annihilation analysis^12^. As the implantation fluence increases to 8.0 × 10^13^ ions/cm^2^, both of emission around 3.20 eV and 2.06 eV enhances dramatically. Meanwhile, strong broad blue emission band centered at 2.84 eV is observed (Figure 6(b)). The dramatic enhancement of light emission can be attributed to the local thermal annealing effect by the electron energy loss during the ion irradiation^24^, which makes the crystallinity of the nanostructures better. Notably, the blue band has not observed in the IL spectra of bulk ZnO under irradiation^9,13^. This indicates that the emission center at 2.84 eV is related with surface defects which is passivated after growth and excited by H^+^ ion irradiation. Absorption of O_2_ or adsorption of -OH on ZnO nanostructure surface would passivate the surface defect emission^35^. ZnO nanorods were grown in solution and the main reactions are as follows^36^:

1 $$Z{n}^{2+}+4O{H}^{-}\to [{(Zn{(OH)}_{4}]}^{2-}$$

2 $$[{(Zn{(OH)}_{4}]}^{2-}\to ZnO+{H}_{2}O+2O{H}^{-}$$

**Figure 7:**
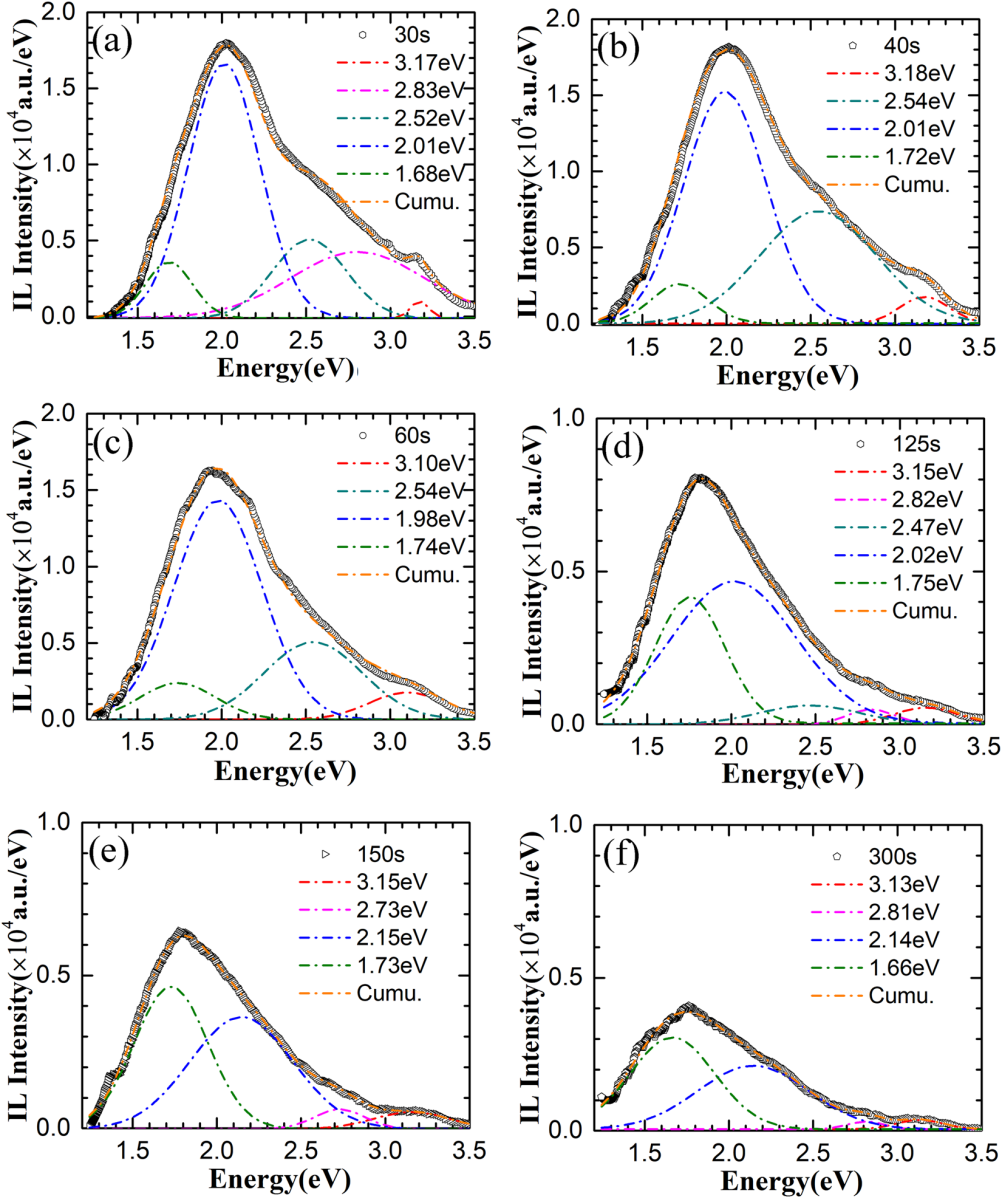
Gaussian decomposition of the real-time IL spectrum of the ZnO nanorods after irradiation by 180 keV H^+^ ions for (**a**) 30 s, (b) 40 s, (**c**) 60 s, (**d**)125 s, (**e**)150 s, (**f**) 300 s, the corresponding H^+^ ions fluences are 4.8 × 10^14^, 6.4 × 10^14^, 9.6 × 10^14^, 1.9 × 10^15^, 2.4 × 10^15^, 4.8 × 10^15^ ions/cm^2^, respectively.

-OH bonds could easily form on the surface of the as-grown ZnO nanorods. -OH bonds could desorb at 150°C and leave Zn dangling bonds^37^. During H^+^ irradiation, the nanorods could be heated locally by energy from H^+^ ions, which would lead to desorption of -OH on the surface, resulting in rapid emergence of blue emission at 2.84 eV related to surface Zn dangling bonds. Meanwhile, desorption of -OH which acting as blind centers could also enhance the total emission as that shown in Fig. 6(b). With a further increase in H^+^ ion fluence to 1.6 × 10^14^ ions/cm^2^, a relatively weak green emission at 2.54 eV is observed in Figure 6(c). The emission center around 2.5 eV has been reported in PL spectra and is ascribed to recombination of a delocalized electron near the conduction band with the deeply trapped hole in *V*_*O*_^16,38–41^. Therefore, the green emission center around 2.54 eV could be ascribed to emission of *Vo*. With a continuous increase in H^+^ ion fluence to 2.40 × 10^14^ ions/cm^2^, all of the emissions at 2.06, 2.84 and 2.54 eV enhance except that the emission at 3.20 eV decreases as shown in Figure 6(d). The weakening of the UV emission is mainly due to the competition of the defect related luminescence. When H^+^ ion fluence increases continuously further, the total emission intensity weakens gradually, but the spectral shape changes as that shown in Figure 7. As the irradiated H^+^ ion fluence increases to 4.8 × 10^14^ ions/cm^2^, new emission centered at 1.68 eV is observed (Figure 7(a)). Emission from 1.65~1.77 eV has been reported in ZnO under ion irradiation and is ascribed to the emission related with single *V*_*Zn*_^13,42^. This means that *V*_*Zn*_ clusters formed at early stage of the irradiation would decompose to single *V*_*Zn*_ under further ion irradiation. With increasing of H^+^ ion fluence to 6.4 × 10^14^ ions/cm^2^, the emission related with *V*_*O*_ is enhanced obviously accompanying with the quenching of blue emission around 2.84 eV and recovery of UV emission at 3.10 eV (Figure 7(b,c)). Emissions at 3.10~3.15 eV are usually attributed to the transition from shallow defects such as *Zn*_*i*_^43^, *H*_*i*_^16^ and *H*_*o*_^7^ to the valence band. Quenching of emission at 2.84 eV could be ascribed to the passivation of surface Zn dangling bonds by out diffused O and H interstitials produced by H^+^ ions, leaving more *V*_*O*_ in the ZnO nanorods which enhances the emission around 2.5 eV. As H^+^ ion fluence increases further, the emission at 1.65~1.74 eV increases, while emission of *V*_*O*_ at 2.45 eV weakens as shown in Fig. 7(d). As H^+^ ion fluence increases to 2.4 × 10^15^ ions/cm^2^, emission around 1.74 eV becomes dominate, accompanying with quenching of green emission and recovery of blue emission around 2.82eV (Fig. 7(e,f)). With a further increase in H^+^ ion fluence, the emission becomes too weak to be investigated in detail. Quenching of green emission has been attributed to the formation of *V*_*O*_*-OH* clusters in ZnO through combination of *V*_*O*_ with H and O interstitials^44^. Then recovery of blue emission around 2.84 eV could be ascribed to incomplete passivation of surface Zn dangling bonds under higher H^+^ ion fluence.

**Figure 8:**
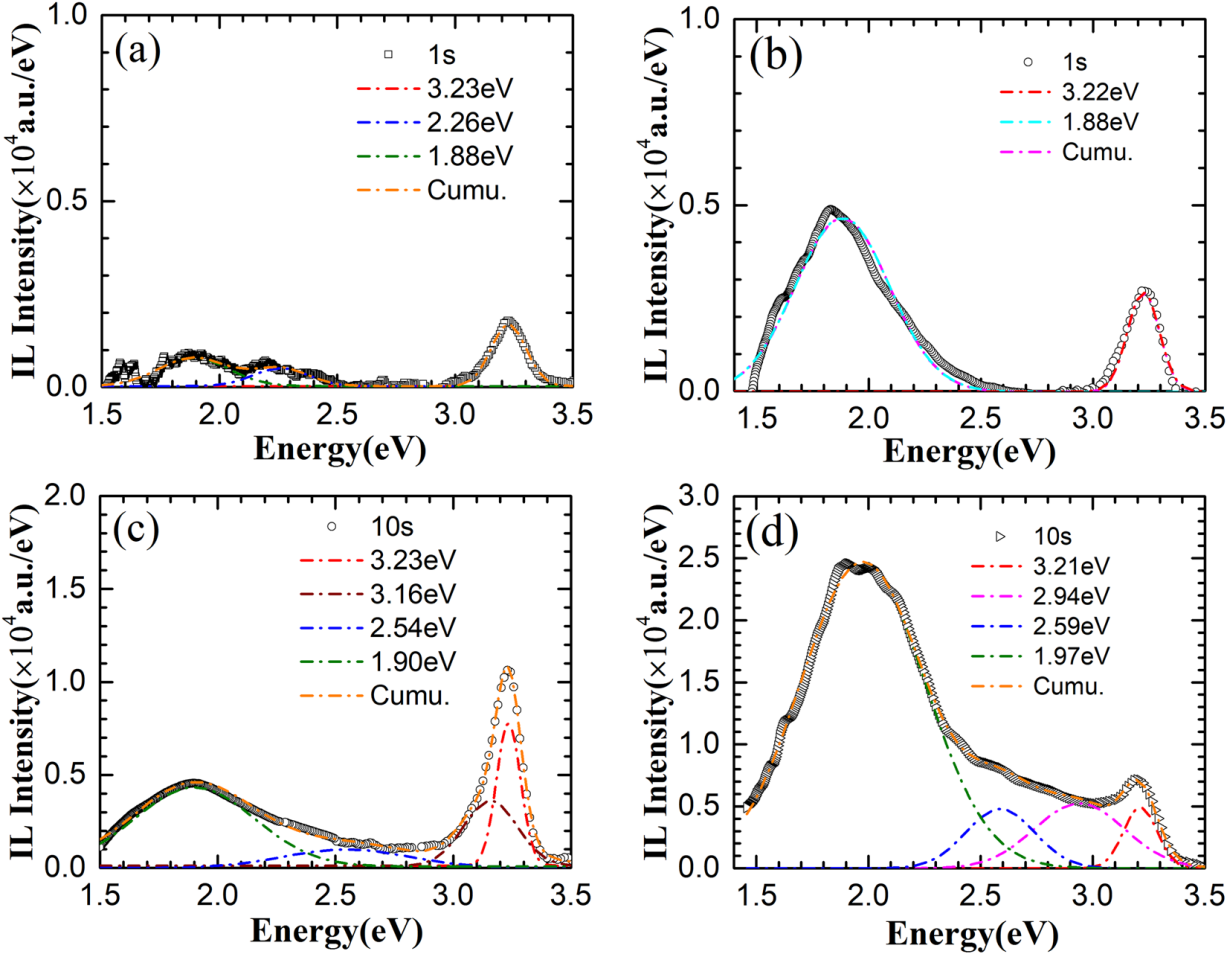
IL spectra of ZnO nanorods passivated in 0.01M C_4_H_7_AlO_5_·2H_2_O solution ((**a,c**)) and annealed in O_2_ at 350 °C for 20 min ((**b,d**)) after irradiation by 180 keV H^+^ ions for 1 s and 10 s, the corresponding H^+^ ions fluences are 1.6 × 10^13^ and 1.6 × 10^14^ ions/cm^2^.

To further confirm the relation of the emission center at 2.84 eV with the nanorod surface states, some of the as-grown ZnO nanorods were passivated in 0.01M C_4_H_7_AlO_5_·2H_2_O solution and the other ones were annealed in O_2_ at 350 °C for 30 min, respectively. For comparison, these ZnO nanorod arrays were irradiated by 180 keV H^+^ under the same conditions with that of the as gown nanorods. Figure 8 show the representative IL spectra of ZnO nanorods passivated with 0.01M C_4_H_7_AlO_5_·2H_2_O solution and IL spectra of ZnO nanorods annealed in O_2_ separately. UV emission at 3.23 eV and weak emission at 1.88 eV and 2.26 eV are observed in surface passivated ZnO nanorods at the beginning of the irradiation (Figure 8(a)). However, as the ion fluence increased to 1.60 × 10^14^ ions/cm^2^, a sharp UV peak and a relatively weak visible emission band can be observed. Gaussian fitting indicates that the luminescence centers locate at 3.23, 3.16, 2.54, and 1.90 eV (Figure 8(c)). The emission at the beginning of the irradiation and after irradiation are much similar to that reported in ZnO bulk^12^, and are different from that of the as-grown ZnO nanorods (Figure 6). This means that the simple surface passivation could improve the radiation resistance of ZnO nanorods. Comparing the IL spectra of the annealed ZnO nanorods with that of unannealed or passivated ones, much stronger visible emission at 1.88 eV and weaker emission at 3.22 eV are observed at the beginning of irradiation (Figure 8(b)). The strong visible emission could be attributed to elimination of non-radiative centers and production of *V*_*Zn*_ related defects after annealing in O_2_ atmosphere. As the ion fluence reaches 1.60 × 10^14^ ions/cm^2^, a significant enhancement of visible emission by 5 times is observed. At the same times, a new blue emission at 2.94 eV and a green emission at 2.59 eV are observed (Figure 8(d)), which are similar to that of the as-grown ones. Appearance of blue emission in IL spectra of both the as-grown and annealed ZnO nanorods and disappearance in the ZnO nanorods passivated in C_4_H_7_AlO_5_·2H_2_O solution after irradiation further confirm that blue emission is related to the surface defects.

### Evolution model of native defects under irradiation

**Figure 9:**
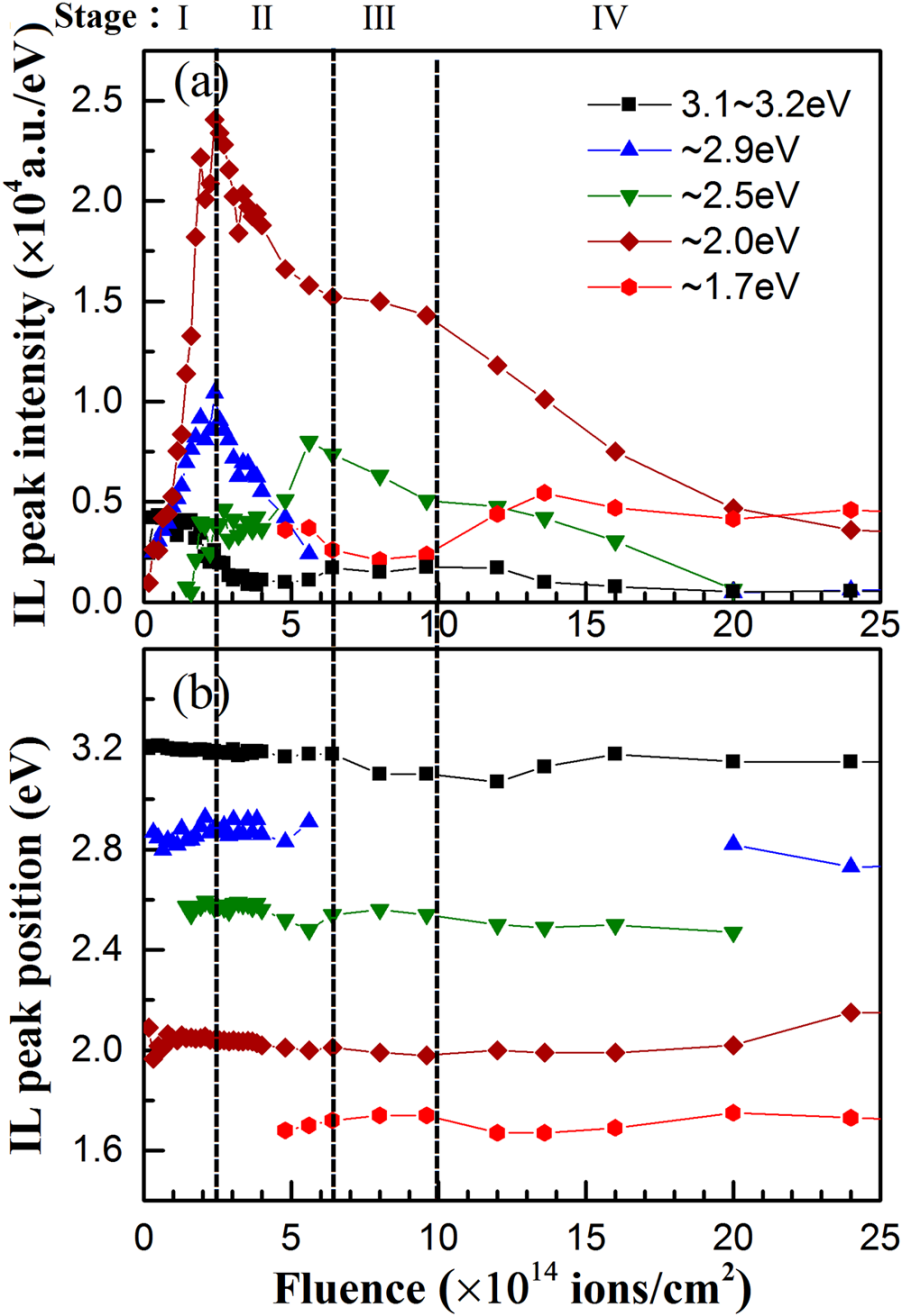
Evolution of (**a**) emission peak intensity and (**b**) peak energy with H^+^ ion fluence derived from Gaussian fitting of the IL spectra of ZnO nanorod arrays irradiated by 180 keV H^+^ ions.

**Figure 10:**
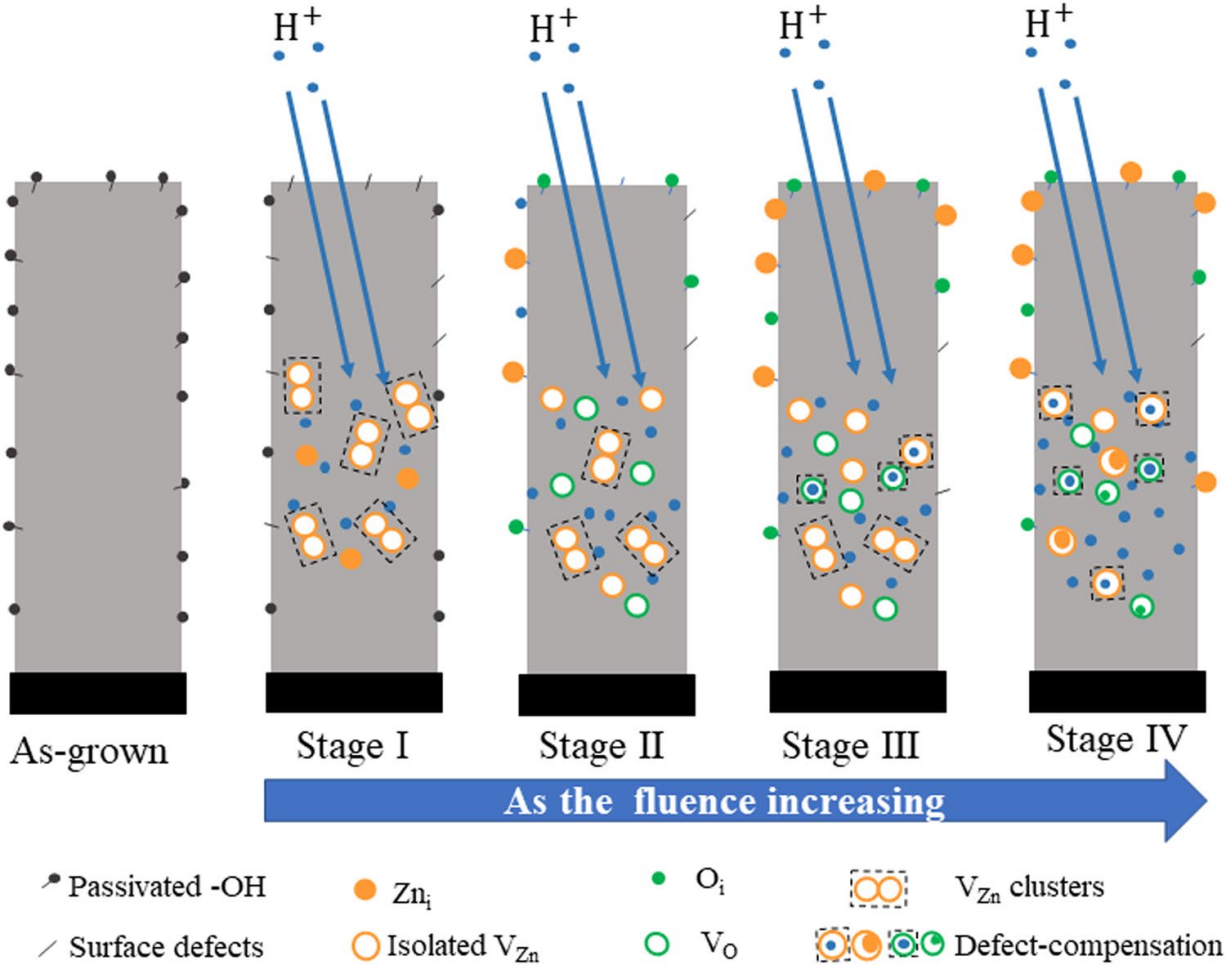
Schematic diagrams indicating the generation, evolution and annihilation of defects in ZnO nanorod arrays under 180 keV H^+^ ion beam irradiation.

From the evolution of the IL spectra from Figure 5–8, it can be seen that the evolution of ZnO nanorod emission is very sensitive to the irradiation fluence. Activation or passivation of UV, blue, green, yellow and red emissions vary obviously with H^+^ ion fluence. To investigate the evolution model of native defects under irradiation, dependence of the emission peak energy and intensity on H^+^ ion fluence are summarized in Figure 9. According to the activation/passivation of different emission centers and variation of the emission intensity, defects evolution under H^+^ ions are divided into four stages as that shown in Figure 10.

Stage I: H^+^ ion fluence varies from 0 to 2.5 × 10^14^ ions/cm^2^. H^+^ ions transfer their energy to ZnO lattices through electronic energy loss process, annealing out the absorbed -OH bonded on the surfaces^38^ and eliminating some of blind centers. Then the light emission enhances and the surface defect emission are excited at initial stage of irradiation. Meanwhile, H^+^ ions transfer their energy to lattice atoms through nuclear energy loss resulting in the displacement of lattice Zn and O atoms. Displacement energy for the Zn and O atoms are 30 and 52 eV, respectively, so *V*_*Zn*_ and *Zn*_*i*_ are the main defects in the initial stage of ion implantation^37^. Therefore, *V*_*Zn*_ clusters are rapidly generated, and their concentration increases significantly with the increasing of H^+^ ion fluence, which result in a very strong emission around 2.0 eV. The emitted light intensity is enhanced dramatically by implanted H^+^ ions.

Stage II: H^+^ ion fluence varies from 2.5 to 6.4 × 10^14^ ions/cm^2^. *Zn*_*i*_, *O*_*i*_ and *H*_*i*_ produced by H^+^ irradiation would diffuse to the nanorod surfaces and passivate the surface defect emission centers due to the easily migration of *Zn*_*i*_, *O*_*i*_ and *H*_*i*_ at 290~325 K^9,45^. Meanwhile, *V*_*Zn*_ and *V*_*O*_ would remain inside the nanorods due to their higher migration energy^4,8^. Otherwise, the migration energy of *O*_*i*_ is much lower than that of *Zn*_*i*_^4^. Then *V*_*O*_ concentration would increase with an increase in H^+^ ion fluence, while some *V*_*Zn*_ clusters would decompose into single *V*_*Zn*_ through combination with newly produced *Zn*_*i*_. Therefore, blue emission around 2.9 eV is quenched quickly and the emission of *V*_*Zn*_ clusters round 2.0 eV would weaken, while the emission of single *V*_*Zn*_ emerges and the emission of *V*_*O*_ are further enhanced.

Stage III: H^+^ ion fluence varies from 6.4 × 10^14^ to 1.0 × 10^15^ ions/cm^2^. Each kinds of defects remains relatively stable. During H^+^ ion irradiation, the existing defects would be compensated by newly produced vacancies and interstitials, thus achieving a relative dynamic balance of defect concentrations. Otherwise, the implanted H atoms could combine with *V*_*O*_ to form *H*_*O*_ complex impurities^17^ or combine with *V*_*Zn*_ to form *V*_*Zn*_-OH clusters which passivate the green emission^21^. Therefore, all emissions keep relatively stable except that the green emission of *Vo*^+^ is weakened.

Stage IV: H^+^ ion fluence is higher than 1.0 × 10^15^ ions/cm^2^. In this stage, more vacancies and interstitials are produced. More *V*_*Zn*_ clusters would combine with newly produced *Zn*_*i*_ and decompose into isolated *V*_*Zn*_ until the concentration reaches a relative stable state. Otherwise more H atoms would combine with *V*_*O*_ to form *H*_*O*_ complex which would passivate green emission related with *V*_*O*_ as shown in Figure 9 and 10. Meanwhile, the blind centers would be introduced which results in the weakening of the total light intensity.

In summary, well-aligned ZnO nanorod arrays were grown on Si substrates in aqueous solution of methenamine and zinc acetate. Then the nanorods were irradiated with 180 keV H^+^ ions to a total fluence of 8.0 × 10^15^ ions/cm^2^. The annealing effect during irradiation would improve the crystallinity of the nanorods. Out-diffusion of the interstitial atoms and the stabilization of H by vacancies would lead to the lattice contraction, which would induce a blue-shift of the UV emission. Evolution of the defects are very sensitive to the irradiated H^+^ ion fluence, especially in the beginning stage. Evolution of the defects under H^+^ ion irradiation could be classified into IV stages. Surface defect emission center round 2.84~2.90 eV could be activated by annealing effects of H^+^ ion and passivated by out diffused interstitials. Green emission of ${V}_{O}$ could be activated by H^+^ ion at the beginning stage of irradiation and be passivated by combination with H^+^ ions as the fluence is higher than 2.0 × 10^15^ ions/cm^2^. *V*_*Zn*_ clusters would form in the beginning stage of irradiation and decompose into isolated *V*_*Zn*_ under higher irradiation. Combination of the existed defects with newly produced ones could perform a relative stable stage of defects. Surface passivation of ZnO nanorods in C_4_H_7_AlO_5_ aqueous solution could improve the irradiation resistance of ZnO nanorods.

## Experimental

### Growth of ZnO nanorod arrays

The ZnO nanorod arrays were grown vertically on Si (100) substrates by the two-step method^19^. Typically, the substrates were cleaned in the ultrasonic bath with acetone, ethanol and deionized water, and then etched by 10% HF solution for 10 min to remove the residual oxide layer. Then 25 nm thick ZnO seed layer was deposited on the substrate by radio frequency magnetron sputtering. Then ZnO nanorod arrays were grown in aqueous solutions of 0.02M Zn(CH_3_COO)_2_·2H_2_O and hexamethylenete-tramine (HMTA) at 95 °C for 2 h. After the growth, the substrates with ZnO nanorods were washed with deionized water, and then dried in a vacuum oven at room temperature. The passivation of ZnO nanorod arrays were performed by immerging the as-grown ZnO nanorods directly in 0.01M aluminium hydroxide acetate (C_4_H_7_AlO_5_·2H_2_O) aqueous solutions for 10 s. Then the nanorods were dried at 75 °C for 5 min. To adjust the shell thickness, the dipping and drying processes were also repeated for 3 times. The annealing process for ZnO nanorod arrays was carried out in oxygen atmosphere with O_2_ flow of 300 sccm at 350 °C for 30 min.

### Irradiation and collection of IL spectra of ZnO nanorod arrays

The ZnO nanorod arrays were irradiated by 180 keV H^+^ with a BNU 400 keV Ion Implanter. The total irradiation fluence is 8 × 10^15^ ions/cm^2^ and the ion flux was 8.02 × 10^13^ ions/cm^2^. During H^+^ ion irradiation, real-time IL spectra were recorded in a chamber with a base pressure of 5 × 10^−4^ Pa connected to the ion implanter. The sample was set in the center of the stage and irradiated continually by 180 keV H^+^ ion beam with an incident angle of 15° with the sample normal direction. The ion beam with a quadrate spot size of 40 × 40 mm^2^ could cover the whole nanorods. A silica lens and an optical fiber were placed at an angle of 32° from the incident H^+^ beam direction to collect the luminescence of IL spectra.

### Characterization of the nanorods before and after irradiation

Surface morphologies and cross sectional images of ZnO nanorods were observed by a field emission scanning electron microscope (FESEM, HITACHI S-4800). The X-ray diffractometer (SHIMADZU XRD-6000) with a Cu Kα radiation was used to investigate the crystal structures of all samples. Photoluminescence (PL) spectra were acquired by a Jobin-Yvon micro-Raman spectrometer using a 325 nm He-Cd laser as an excitation source.
